# Defect Synergistic Regulations of Li&Na Co‐Doped Flexible Cu_2_ZnSn(S,Se)_4_ Solar Cells Achieving over 10% Certified Efficiency

**DOI:** 10.1002/advs.202306740

**Published:** 2023-12-06

**Authors:** Quanzhen Sun, Chen Shi, Weihao Xie, Yifan Li, Caixia Zhang, Jionghua Wu, Qiao Zheng, Hui Deng, Shuying Cheng

**Affiliations:** ^1^ Institute of Micro‐Nano Devices and Solar Cells College of Physics and Information Engineering Fuzhou University Fuzhou 350108 P. R. China; ^2^ Fujian Science & Technology Innovation Laboratory for Optoelectronic Information of China Fuzhou Fujian 350108 P. R. China; ^3^ Jiangsu Collaborative Innovation Center of Photovoltaic Science and Engineering Changzhou 213164 P. R. China

**Keywords:** certified efficiency, defect regulations, flexible CZTSSe solar cells, large‐area flexible device, Li&Na co‐doping

## Abstract

Ion doping is an effective strategy for achieving high‐performance flexible Cu_2_ZnSn(S,Se)_4_ (CZTSSe) solar cells by defect regulations. Here, a Li&Na co‐doped strategy is applied to synergistically regulate defects in CZTSSe bulks. The quality absorbers with the uniformly distributed Li and Na elements are obtained using the solution method, where the acetates (LiAc and NaAc) are as additives. The concentration of the harmful Cu_Zn_ anti‐site defects is decreased by 8.13% after Li incorporation, and that of the benign Na_Zn_ defects is increased by 36.91% after Na incorporation. Synergistic Li&Na co‐doping enhances the carrier concentration and reduces the interfacial defects concentration by one order of magnitude. As a result, the flexible CZTSSe solar cell achieves a power conversion efficiency (*PCE*) of 10.53% with certified 10.12%. Because of the high *PCE* and the homogeneous property, the Li&Na co‐doped device is fabricated to a large area (2.38 cm^2^) and obtains 9.41% *PCE*. The co‐doping investigation to synergistically regulate defects provides a new perspective for efficient flexible CZTSSe solar cells.

## Graphical abstract

The Li&Na co‐doped strategy is applied to synergistically regulate defects in CZTSSe bulks using the cheap and non‐toxic LiAc and NaAc as additives. The synergistic co‐doping effectively passivates harmful defects and increases shallow‐level defects. The flexible CZTSSe device achieves 10.53% efficiency (certified 10.12%) in the area (0.21 cm^2^) and 9.41% efficiency in large area (2.38 cm^2^).

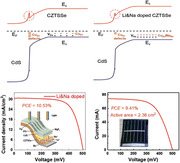

## 1 Introduction

Flexible thin‐film solar cells show great potential applications in weight‐restriction places, curved surfaces, wearable and portable electronics, solar‐powered autos, and so on.^[^ ^1^, ^2^, ^3^, ^4^, ^5^ ^]^ Cu_2_ZnSn(S,Se)_4_ (CZTSSe) has become a great potential absorber material due to its low cost, earth‐abundant and environmental elemental components, and stable structure.^[^ ^3^, ^6^, ^7^, ^8^, ^9^, ^10^ ^]^ The highest power conversion efficiency (*PCE*) of the flexible CZTSSe solar cell has exceeded 12.84%,^[^ ^11^ ^]^ but it is still lower than that (20.8%) of the flexible CIGS solar cell.^[^ ^12^ ^]^ The large open‐circuit voltage deficit (*V*_oc,def_, *E*_g_/*q*–*V*_oc_) and relatively low fill factor (*FF*) are the key issues limiting the flexible CZTSSe solar cell performance.^[^ ^13^, ^14^, ^15^ ^]^

The relevant reports showed that interface recombination, band tailing, deep‐state defects, and the unsatisfactory crystallinity of the absorber may be responsible for the large *V*_oc, def,_ and low *FF* in the kesterite CZTSSe solar cells.^[^ ^16^, ^17^, ^18^, ^19^ ^]^ Alkali metal (Li, Na, K, and so on) cation doping is one of the most promising strategies to solve those problems by defect passivation and crystallization promotion of the CZTSSe films. For the alkali metal ions, Li is likely to be incorporated into the CZTSSe lattice to reduce defect states due to its smaller ionic radius close to that of the Cu^+^ and the low substitution energy of Li_Cu_. In recent years, Li doping techniques have been proven to enhance the performance of CZTSSe solar cells.^[^ ^20^, ^21^ ^]^ Zhang et al. developed a Se&LiF selenization technology to fulfill a simple and efficient Li‐doping and engineer the band alignment of the CZTSSe/CdS interface, achieving an encouraging device PCE of 11.63%.^[^ ^22^ ^]^ Subsequently, they introduced Li and S elements into the CZTSe film by Li_2_S as raw material to passivate defects of the CZTSSe layer, which increased the *V*_oc_ of the device by 120 mV.^[^ ^23^ ^]^ Cabas‐Vidani et al. successfully prepared a Li‐alloyed (Li_x_Cu_1‐x_)_2_ZnSn(S,Se)_4_ absorber and achieved the champion device *PCE* of 11.6% because of the widened bandgap and decreased *V*_oc,def_.^[^ ^24^ ^]^ Meng et al. reduced interface defects and achieved a certified device PCE of 12.7% due to the better band bending that was realized by incorporating Li into the CZTSSe absorber.^[^ ^25^ ^]^ The above studies on Li doping were all conducted in the rigid CZTSSe solar cells based on sodium‐rich soda‐lime glass (SLG) substrates, where they all showed Li ions can facilitate the crystal growth of the CZTSSe absorbers. However, Pan et al. found that Li ions had little effect on crystal growth by studying the CZTSSe absorbers prepared on quartz glass (without Na) substrates.^[^ ^26^ ^]^ Subsequently, they discovered the Li/Na exchange mechanism, where Li ions can induce Na in the SLG substrates diffusing into CZTSSe rather than entering the host lattice to form Li‐alloyed CZTSSe films.^[^ ^27^ ^]^ The heavier alkali elements, typically Na, can significantly enhance the grain size and passivate the defects of grain boundaries for the CZTSSe absorber.^[^ ^28^ ^]^ Therefore, the grain growth could be the effect of Na on the CZTSSe films in the Li‐doped CZTSSe devices based on SLG substrates. Moreover, due to the presence of Na in SLG substrates, it is difficult to study the influence of single Li doping on the CZTSSe characteristics in rigid solar cells.

Flexible CZTSSe solar cells based on Mo foils showed lower efficiency because of the absence of Na in the substrates.^[^ ^29^, ^30^, ^31^ ^]^ Researchers introduced Na elements into the substrate or CZTSSe absorber to improve the *PCE*s exceeding 9.63%.^[^ ^32^ ^]^ Our group has proven that Li ions can enhance carrier transport and achieved the device *PCE* of 9.68%.^[^ ^33^ ^]^ In CZTSSe film, both the Li and Na ions played a positive role in enhancing the carrier concentration.^[^ ^34^, ^35^ ^]^ Therefore, the favorable synergistic effects between Li and Na in defect passivation, grain growth, and carrier concentration enhancement are expected to enhance the *PCEs* of the flexible CZTSSe solar cells. Due to the absence of alkali metal ion in the Mo foils, the detailed mechanisms of Li doping and Na doping are expected to be studied independently and systematically in the flexible solar cells.

In this work, we prepare Li&Na co‐doped flexible CZTSSe solar cells using the solution method. The 10.53% *PCE* (certified 10.12%, 0.2025 cm^2^) and 9.41% large‐area *PCE* (2.38 cm^2^) of flexible CZTSSe devices have been achieved. The influence of alkali metals doping on CZTSSe films and their device performances are explored by systematic measurements such as temperature‐dependent conductivity (TDC), admittance spectroscopy (AS), the capacitance‐voltage (*C*‐*V*), and drive level capacitance profiling (DLCP). It indicates that Li incorporation decreases harmful defects and Na incorporation increases shallow defects. Moreover, both the Li and Na incorporations enhance carrier concentration and reduce the interfacial defects. By defect synergistic regulations of Li and Na co‐doping, the average *V*_oc_ of the devices realizes a significant improvement of 21.71%.

## 2 Results and Discussion

### 2.1 Design of Li&Na Co‐Doped Flexible CZTSSe Solar Cells

**Figure 1:**
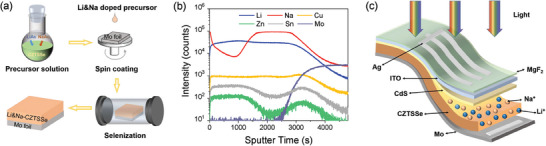
a) Schematic diagram of Li&Na doping approach in CZTSSe absorbers. b) SIMS depth profile of the Li&Na co‐doped CZTSSe film. c) Device structure of the flexible CZTSSe solar cell based on Mo foil.

The Li and Na ions doping are realized by the solution method (**Figure** **1**a), in which the LiAc and NaAc are joined in the CZTSSe precursor solution. The distributions of metallic elements in the Li& Na co‐doped CZTSSe film are analyzed by time‐of‐flight secondary ion mass spectroscopy (TOF‐SIMS) as shown in Figure 1b. The Li element is uniformly distributed throughout the absorber while the signal of the Na element near the surface is slightly weaker than that in the bulk. It demonstrates that the doped ions are accurately controlled and uniformly dispersed in the absorber. The optimal conditions for alkali metal ions doping are determined by the *PCE* statistics (Figure S1, Supporting Information) of the flexible CZTSSe solar cells (Figure 1c) with different Li/(Cu+Zn+Sn) and Na/(Cu+Zn+Sn) ratios. The optimal values of Li and Na contents are 10% and 1% (Figure S1a,b, Supporting Information), respectively. Figure S1c (Supporting Information) shows the optimal *PCE*s of flexible devices with Li of 10% and Na of 1%, which are higher than that of the single‐alkali metal‐doped flexible devices. The optimal doping contents are used to further investigate the improvement mechanism of device performance, named Li‐doped, Na‐doped, and Li&Na co‐doped devices.

### 2.2 Characterizations of CZTSSe Films

**Figure 2:**
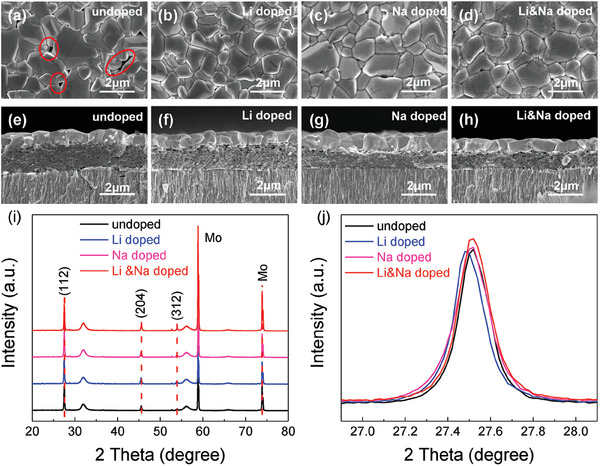
a–d) Top‐view SEM images and e–h) cross‐section SEM images of undoped, Li‐doped, Na‐doped, and Li&Na co‐doped CZTSSe absorbers. The XRD patterns (i) and magnified views of (112) peaks (j) for different absorbers.

**Figure** **2**a–h displays the top‐view and cross‐sectional scanning electron microscope (SEM) images of the four CZTSSe films, respectively. The alkali metal‐doped CZTSSe films (Figure 2b–d) are more uniform and denser compared with the undoped CZTSSe film (Figure 2a). Furthermore, white granules appear on the surface of the Na‐doped and Li&Na‐doped CZTSSe films (Figure 2c,d). According to the related report,^[^ ^27^ ^]^ the white granules could be some Na compounds. That may be formed because the superficial Na element moves to the film surface during selenization, explaining the weaker signal of Na element near the surface of Li&Na doped CZTSSe film (Figure 1b). These Na compounds would be eliminated during the deposition of the CdS layer because they are soluble in water. There are a few differences in the cross‐sectional morphologies for the undoped and Li‐doped CZTSSe films (Figure 2e,f). Whereas the fine‐grain layer is significantly thinner with the introduction of Na (Figure 2g,h). Figure 2i shows the X‐ray diffraction (XRD) patterns of the films. All the films display similar XRD peaks of (112), (204) and (312), corresponding with the XRD patterns of the kesterite tetragonal phase.^[^ ^36^, ^37^ ^]^ Figure 2j shows the enlarged view of preferentially oriented (112) peaks to explore the effect of alkali metal ions doping on the XRD peak position of the CZTSSe films. Compared with the undoped CZTSSe film, the (112) peak position of Li‐doped CZTSSe film shifts toward a smaller 2*θ* value while that of Na‐doped and Li&Na co‐doped CZTSSe films remain unchanged, indicating Li ions are incorporated into the CZTSSe host lattices to substitute Cu ions in the absence of Na.^[^ ^24^, ^38^ ^]^

The compositions of different CZTSSe absorbers were analyzed by an energy dispersive spectrometer (EDS) and the results are summarized in Table S1 (Supporting Information). The Na contents are almost 0 in undoped and Li‐doped CZTSSe. The Na contents in Na‐doped and Li&Na co‐doped CZTSSe absorbers and Li&Na co‐doped CZTSSe precursor film are 0.0090, 0.0103 and 0.0105, which are consistent with the Na content in the precursor solutions. That indicates that there are almost no loss of Na content before and after selenization. Moreover, the S/(S+Se) rates of Li‐doped and Li&Na co‐doped CZTSSe absorbers are 0.0775 and 0.0884, respectively, which are significantly higher than that of undoped and Na‐doped CZTSSe absorbers. It indicates that Li‐doping can increase the S content in absorbers.

### 2.3 Performances of Flexible Devices

**Figure 3:**
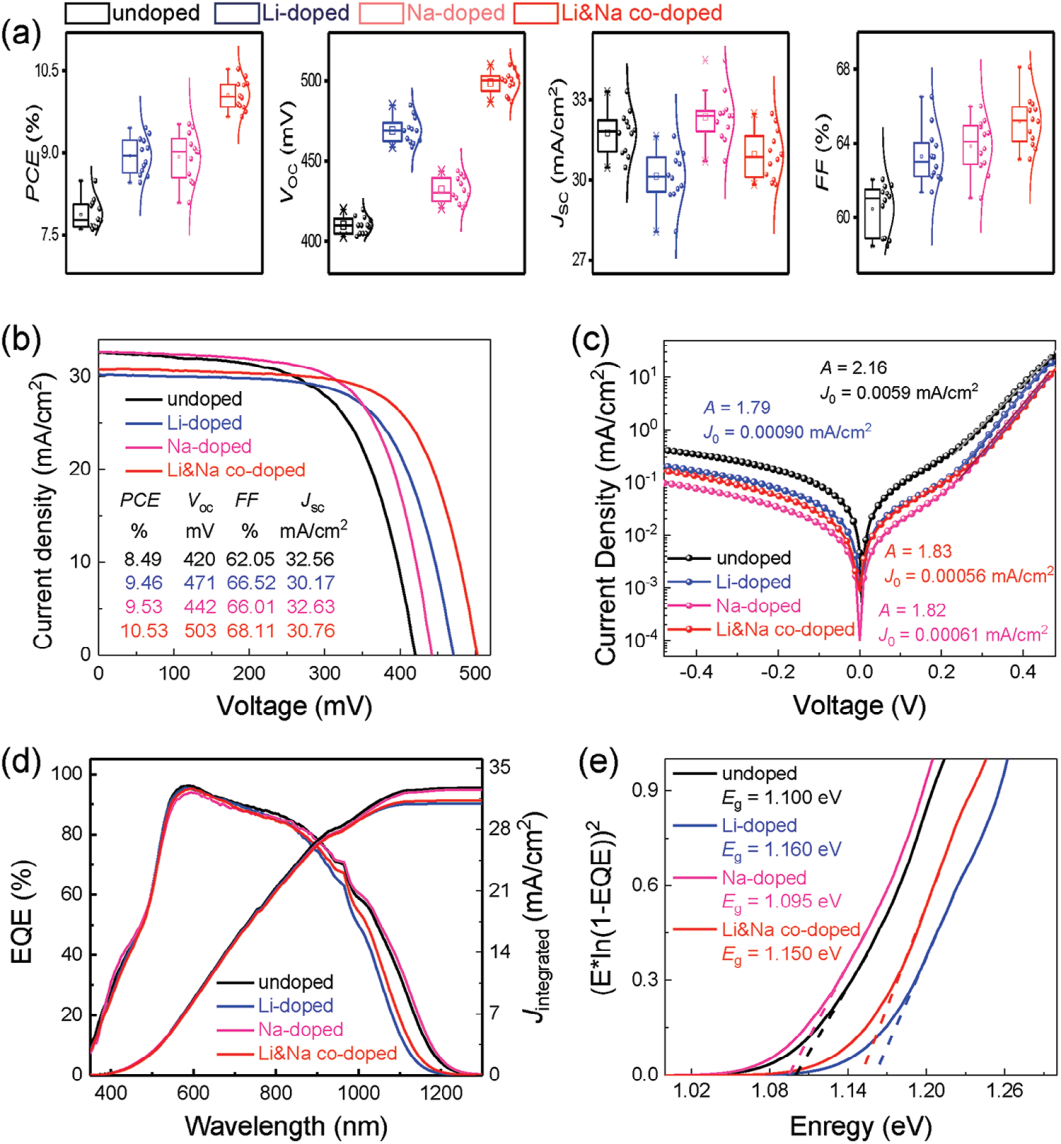
a) Statistical performance parameter box plots of solar cells. The sample size is 12 for each group. b) Light and c) dark *J‐V* characteristic curves of flexible devices. d) The EQE spectra of four devices. e) The *E*_g_ of four absorbers extracted from the EQE data.

The different flexible solar cells are fabricated to investigate the effect of alkali metal ions doping on the device performances. **Figure** **3**a shows the statistical photovoltaic parameters of four flexible devices, which are summarized in Table S2 (Supporting Information). The performance parameters of the alkali metal‐doped devices, especially Li&Na co‐doped devices, have been obviously improved. Compared with the undoped devices, the average *PCE*, *V*_oc,_ and *FF* values of Li&Na co‐doped devices obviously increase by 27.83%, 21.71%, and 7.81%, respectively. For the short circuit current density (*J*_sc_), the average values of Li‐doped devices decreased by 5.13%, while that of Na‐doped devices increased by 1.86%. Ultimately, the average *J*_sc_ value of Li&Na co‐doped devices decreased by 2.58%. It indicates that the reduction of device *J*_sc_ is attributed to Li doping.

The current density‐voltage (*J‐V*) characteristic curves and detailed device parameters of the best devices are illustrated in Figure 3b. The Li&Na co‐doping is more effective than the single alkali metal (Li or Na) doping for the performance improvement of flexible CZTSSe devices with the device *PCE* increased from 8.49% to 10.53%. Simultaneously, the certified *PCE* of 10.12% (active area: 0.2025 cm^2^) for the Li&Na co‐doped flexible CZTSSe solar cell has been achieved (Figure S2, Supporting Information), which is certified by National PV Industry Measurement and Testing Center (NPVM) at Fujian Metrology Institute. In addition, the *V*_oc_ and *FF* are significantly increased from 420 mV and 62.05% to 503 mV and 68.11%, respectively, which are the highest *V*_oc_ and *FF* values for the flexible CZTSSe solar cells prepared by the non‐hydrazine solution method. The diode parameters of the devices extracted from the *J*‐*V* data are summarized in **Table** **1**. The ideality factor (*A*) and reverse saturation current density (*J*_0_) are extracted from the dark *J*‐*V* data of devices (Figure 3c). The series resistance (*R*_s_), shunt conductance (*G*_sh_), and *A* are the important factors affecting the *FF* of the devices.^[^ ^39^ ^]^ The *R*_s_ values of the flexible devices are barely different, while the *G*_sh_ and *A* values are decreased after alkali metal doping. The decrease of *G*_sh_ and the more desirable *A* are the main reasons for the *FF* improvement of alkali metal‐doped devices, indicating the quality junction has been achieved.^[^ ^40^ ^]^ Notably, the *G*_sh_ of the device is reduced from 3.72 to 1.82 and 2.07 mS cm^−2^ after Li doping and Na doping, while that is further decreased to 0.19 mS cm^−2^ after Li&Na co‐doping. The synergistic effect of Li&Na co‐doping in terms of *G*_sh_ significantly increases the *FF* of the device from 62.05% to 68.11%. The *J*_0_ values of alkali metal‐doped devices are all reduced an order of magnitude (decreasing from 10^−3^ to 10^−4^ mA cm^−2^), showing theless recombination in the depletion region and the high‐quality interface.

**Table 1:** The diode parameters and *V*_oc,def_ of four flexible CZTSSe solar cells.

Solar cells	*G*_sh_ [mS cm^−2^]	*R*_s_ [Ω·cm^2^]	*E*_g_/*q*‐*V*_oc_ [mV]
Undoped	3.72	0.51	680
Li‐doped	1.82	0.59	689
Na‐doped	2.07	0.56	653
Li&Na co‐doped	0.19	0.62	647

Figure 3d shows the external quantum efficiency (EQE) spectra of the devices. The integrated *J*_sc_ of the undoped, Li‐doped, Na‐doped, and Li&Na co‐doped flexible CZTSSe solar cells are 32.73, 30.93, 32.55, and 31.26 mA cm^−2^, which are consistent with *J*_sc_ in the *J*‐*V* data. From the *E*_g_ values extracted from EQE data (Figure 3e), it is found that the *E*_g_ of the absorber increases from 1.10 to 1.16 eV as Li is introduced into CZTSSe film, while that of Na‐doped CZTSSe absorber is slightly lower than that of the undoped CZTSSe absorber. As a result, the *E*_g_ of Li&Na co‐doped CZTSSe absorber is 0.01 eV lower than that of the Li‐doped CZTSSe absorber. It demonstrates that Li doping can widen the *E*_g_ of the CZTSSe absorber. The *V*_oc,def_ values of undoped, Li‐doped, Na‐doped and Li&Na co‐doped devices are 680, 689, 653, and 647 mV, respectively. It manifests that Na doping can effectively reduce the *V*_oc,def_. The widened *E*_g_ and reduced *V*_oc,def_ all contribute to the improvement of device *V*_oc_. Benefiting from the synergistic effect of Li and Na doping, the V of the flexible device is significantly increased to 503 mV.

### 2.4 Recombination Mechanism and Defect Properties of Flexible Devices

**Figure 4:**
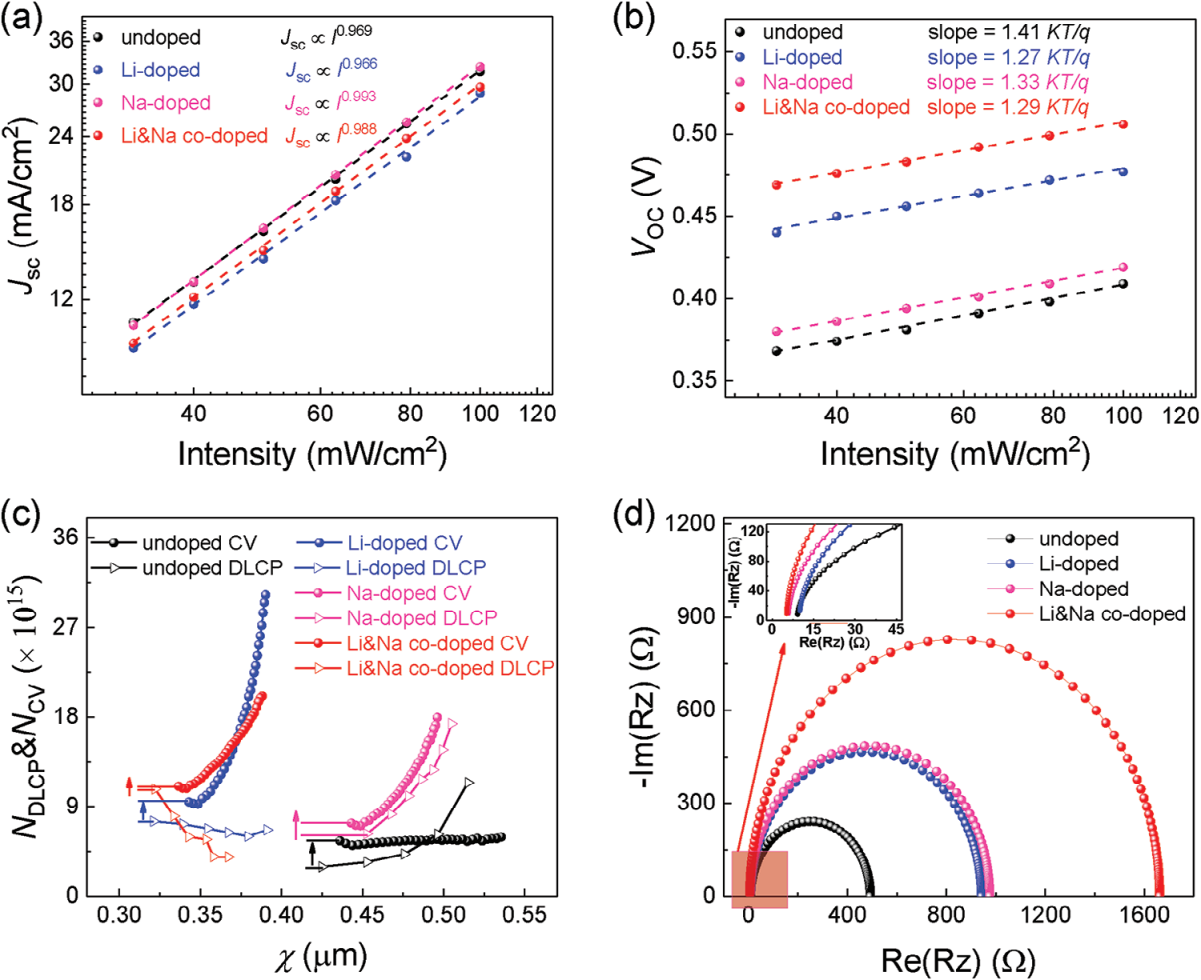
a) The *J*_sc_ and b) *V*_oc_ versus light intensity plots of the four devices. c) The *C*‐*V* and DLCP characteristics of undoped, Li‐doped, Na‐doped, and Li&Na co‐doped flexible CZTSSe devices. d) EIS Nyquist plots of the four flexible devices at a 0.3 V bias.

The *J*_sc_ and *V*_oc_ depend on light intensity (*I*_light_) characteristics and are studied to analyze the recombination mechanism in the devices. **Figure** **4**a shows the power law relationship between *J*_sc_ and *I*_light_ (*J*_sc_ ∝ *I*_light_ *^α^*) of the devices. The *α* values of undoped, Li‐doped, Na‐doped, and Li&Na co‐doped devices are 0.969, 0.966, 0.993, and 0.988, respectively. The *α* values are close to 1, indicating that trap‐assisted recombination is dominant in devices.^[^ ^41^ ^]^ Moreover, the larger *α* value uncovers an enhanced carrier extraction capability in the Na‐doped and Li&Na co‐doped flexible solar cells.^[^ ^42^ ^]^ The *I*_light_‐dependent *V*_oc_ for four devices is shown in Figure 4b. The specific value (*n*) between the slope of *V*_oc_ versus ln(*I*_light_) and (*kT/q*) is 1 in trap‐free solar cells.^[^ ^43^ ^]^ The *n* values for undoped, Li‐doped, Na‐doped, and Li&Na co‐doped devices are 1.41, 1.27, 1.33, and 1.29, respectively. The decreased *n* values indicate the lessened trap‐assisted recombination in alkali metal‐doped devices.^[^ ^44^ ^]^ Moreover, there is the lowest *n* value in the Li‐doped device, demonstrating that Li doping has a better inhibition effect on trap‐assisted recombination in devices.

The interfacial defect states of the devices are explored by the *C*‐*V* and DLCP characteristics (Figure 4c), whose results are summarized in **Table** **2**. The *N*_CV_ is sensitive to both bulk carrier density and interface traps while the *N*_DLCP_ is only sensitive to bulk carrier density.^[^ ^45^ ^]^ Therefore, the difference (*N*_IT_) between *N*_CV_ and *N*_DLCP_ at zero bias can assess the interfacial defects of devices. The *N*_IT_ values of undoped, Li‐doped, Na‐doped, and Li&Na co‐doped devices are 2.60 × 10^15^, 1.92 × 10^15^, 1.08 × 10^15^, and 2.00 × 10^14^ cm^−3^, respectively. The results indicate that both Li doping and Na doping can reduce interfacial defects. The combined action of Li doping and Na doping is more effective for the inhibition of the interface defects, which would significantly reduce interfacial recombination. Moreover, the *N*_CV_ values of alkali metal‐doped flexible devices are all higher than those of the undoped flexible device, demonstrating both Li doping and Na doping can increase the carrier concentration. That is beneficial for the *V*_oc_ enhancement of devices. Figure 4d exhibits the electrochemical impedance spectroscopy (EIS) to better understand the interface information and carrier transport behaviors of the flexible devices. The recombination resistance (*R*_re_) obviously increases from 484 to 1657 Ω after Li&Na co‐doping, which is consistent with less recombination and an elevated *V*_oc_. In addition, the decreased series resistance (*R*_0_) (from 9.06 and 9.84 to 5.76 and 5.53 Ω) of Na‐doped and Li&Na co‐doped flexible CZTSSe solar cells further supports the enhancement of charge transport capacity that is observed in the *I*_light_ dependent *J*_sc_ measurement.

**Table 2:** The results derived from the *C*‐*V* and DLCP measurements at zero bias.

Solar cell	*N*_CV_ [cm^−3^]	*N*_DLCP_ [cm^−3^]	Interface state [cm^−3^]
undoped	5.56 × 10^15^	2.96 × 10^15^	2.60 × 10^15^
Li‐doped	9.47 × 10^15^	7.55 × 10^15^	1.92 × 10^15^
Na‐doped	7.36 × 10^15^	6.28 × 10^15^	1.08 × 10^15^
Li&Na co‐doped	1.10 × 10^16^	1.08 × 10^16^	2.00 × 10^14^

The bulk defect properties of the absorbers are determined by the TDC characteristic acquiring the *I*‐*V* curves of the four films in the temperature range of 100–320 K (Figure S4a–c, Supporting Information). It is measured by a structure of Au/absorber/Au prepared on the quartz glasses (Figure S3, Supporting Information). At higher temperatures, the conduction type of p‐type semiconductor carrier is mainly the thermally activated band conduction.^[^ ^46^ ^]^ As the temperature drops, most of the holes are recaptured by the acceptors in the semiconductor, which cannot jump directly to the valence band due to the lack of energy. Consequently, the holes will be conducted through the impurity band, where the nearest neighbor hopping (NNH) conduction is the primary conduction mechanism.^[^ ^47^ ^]^ The conductivity can be described as Equation (1). At the lower temperature, the holes in the semiconductor hop between the levels close to the Fermi level, where Mott's 3D variable range hopping (Mott's VRH) becomes the primary conduction mechanism. The conductivity can be described as Equation (2).^[^ ^48^ ^]^

(1) $$\sigma \;=\sigma_{0}\;exp\left(-\frac{E_{1}}{kT}\right)\;+\;\sigma_{0N}exp\left(-\frac{E_{2}}{kT}\right)$$

(2) $$\sigma \;=\sigma_{0M}\;T^{-1/2}\exp\left[-{\left(\frac{T_{M}}{T}\right)}^{1/4}\right]$$

**Figure 5:**
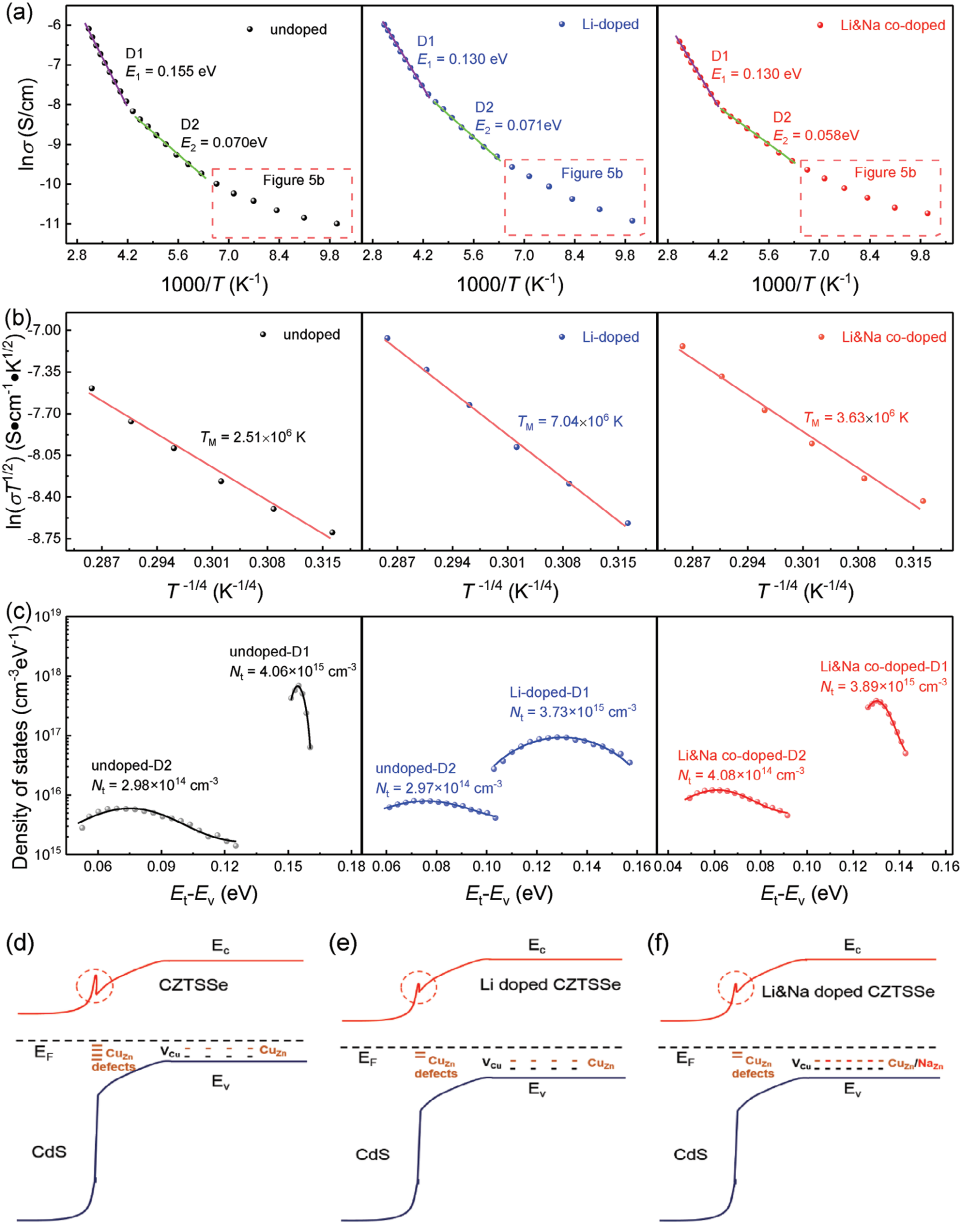
a) Arrhenius plots of conductivity, b) the plots of ln(σT^1/2^) over T^−1/4^, and c) the defect distributions of D1 and D2 for undoped, Li‐doped and Li&Na co‐doped CZTSSe films. Schematic diagram of band and acceptors variation of d) undoped, e) Li‐doped and f) Li&Na co‐doped flexible CZTSSe solar cells.

Where, *σ* is the conductivity, *σ*_0_, *σ*_0N_ and *σ*_0M_ are the pre‐exponential factor that is proportional to the grain size and average carrier concentration and independent of *T*, *k* is the Boltzmann constant, *T* is the temperature, *E*_1_ is the activation energy, *E*_2_ is the nearest neighbor activation energy, and *T*_M_ is the Mott's temperature. **Figure** **5**a shows the plots of ln*σ* versus 1000/*T* for the films from Equation (1). There are two kinds of defects in all three absorbers, named D1 and D2, respectively. It can be seen that the *E*_1_ of D1 decreases from 0.155 to 0.130 eV after Li doping while that of D1 remains the same after the Na is introduced into the Li‐doped CZTSSe film. It indicates that Li doping leads to a decrease in the *E*_1_ of D1 in the CZTSSe film. According to the reported investigations,^[^ ^33^, ^49^, ^50^ ^]^ the Cu_Zn_ anti‐site defects have a defect level of 0.130–150 eV. Therefore, the deeper D1 (0.155 eV) of the CZTSSe film should be ascribed to Cu_Zn_ anti‐site defects, which will act as recombination centers to capture photogenerated carriers and thus worsen device performance.^[^ ^14^, ^51^ ^]^ According to the first‐principles calculation,^[^ ^52^ ^]^ Li ions are likely to replace the positions of Cu ions to form shallower Li_Zn_ defects due to the radius close to that of Cu^+^ and the low substitution energy, which may be the cause for the decrease of *E*_1_. The *E*_2_ values of D2 in the undoped, Li‐doped, and Li&Na co‐doped CZTSSe films are 0.070, 0.071, and 0.058 eV, respectively. It demonstrates that the Na incorporation leads to a decrease in the *E*_2_ of D2 for the CZTSSe film. According to the theoretical investigation,^[^ ^53^, ^54^ ^]^ the D2 with *E*_2_ of ≈0.070 meV can be assigned as Cu vacancy (V_Cu_) defects, and the Na_Zn_ defects to contribute holes for the material can be formed due to its low formation energy. The formation of shallow acceptor Na_Zn_ defects is equivalent to increasing the Cu vacancy (V_Cu_) in the material, which is conducive to the improvement of device performance. Figure 5b shows the plots of ln(*σT* ^1/2^) over *T* ^−1/4^ for the films, where the *T*_M_ is obtained by fitting the linear. The *T*_M_ is a characteristic temperature related to the disorder of the film material.^[^ ^47^ ^]^ The higher *T*_M_ value indicates a higher disorder in the film. It can be observed that the *T*_M_ value of CZTSSe film enhances from 2.51 × 10^6^ to 7.04 × 10^6^ K after Li doping. Therefore, the CZTSSe film becomes more disordered after Li doping, which may be attributed to the similar radii of Li, Cu, and Zn ions. Moreover, the *T*_M_ is inversely proportional to the density of states at the Fermi level (*T*_M_ ∝ [*N*(E_F_)]^−1^).^[^ ^55^ ^]^ Consequently, Li doping decreases energy states at the Fermi level of the CZTSSe films. According to related reports,^[^ ^48^ ^]^ the decrease in energy states at the Fermi level implies the reduction in the Cu_Zn_ anti‐site defects. Thus, Li doping effectively reduces the *N*_t_ of Cu_Zn_ anti‐site defects. However, the *T*_M_ value is restored to 3.63 × 10^6^ K after Na is introduced into the Li‐doped CZTSSe film, which may be attributed to Na ions preventing Li ions from entering the CZTSSe master lattice.

The defect density (*N*_t_) is extracted from admittance spectroscopy (AS) (Figure S4d‐f, Supporting Information) by using T. Walter's method^[^ ^56^, ^57^ ^]^ to further explore the effect of alkali metal doping on the bulk defects of the CZTSSe films. The formulas related to AS are as follows^[^ ^58^ ^]^:

(3) $$\omega_{0}=\;2\upsilon_{0}exp\left(-\frac{E_{a}}{kT}\right)$$

(4) $$E\left(\omega \right)\;=\;kTln\left(\frac{2\upsilon_{0}}{\omega }\right)$$

(5) $$N_{t}\left(\omega \right)\;=\;-\frac{V_{d}}{qW}\cdot \frac{dC}{d\omega }\cdot \frac{\omega }{kT}$$

Where *ω*_0_ is the inflection angular frequency of the electronic transition, υ_0_ is the attempt‐to‐escape frequency, *E* is the energy of defects concerning the VBM, *ω* is the angular frequency, *N*_t_(*ω*) is the density of defect states, *V*_d_ is the built‐in voltage and *W* is the depletion region width. Figure 5c shows the defect state distributions of D1 and D2 for undoped, Li‐doped and Li&Na co‐doped flexible CZTSSe solar cells. The *N*_t_ of D1 (Cu_Zn_ defects) for the absorber decreases from 4.06 × 10^15^ to 3.73 × 10^15^ cm^−3^ after Li doping, while that of D1 defect is slightly elevated to 3.89 × 10^15^ cm^−3^ when Na is introduced to Li‐doped CZTSSe absorber. The decreased *N*_t_ demonstrates that Li doping inhibits harmful Cu_Zn_ defects, which reduces trap‐assisted recombination. For the D2 defect, the *N*_t_ values of undoped and Li‐doped CZTSSe absorbers are 2.98 × 10^14^ and 2.97 × 10^14^ cm^−3^, whereas that (4.08 × 10^14^ cm^−3^) of Li&Na co‐doped CZTSSe absorber is higher 36.91% than that of undoped CZTSSe film. It shows that Na doping increases the shallow acceptor defects. Therefore, in the Li&Na co‐doped CZTSSe film, Li doping reduces harmful Cu_Zn_ defects and Na doping increases the Na_Zn_ shallow defects. The defect synergistic regulations by Li&Na co‐doping effectively decrease the recombination and enhance the carrier concentration, greatly improving the device performance.

The variation of bands and acceptors for three flexible devices are shown in Figure 5d–f. As for the CZTSSe solar cell, at CdS/CZTSSe interface, the p‐type Cu_Zn_ defects with high concentration pin the Fermi level (E_F_) in the middle of the bandgap,^[^ ^50^ ^]^ and the conduction band offset (CBO) is spike‐type (Figure 5d). The small spike‐type CBO is beneficial in suppressing interfacial recombination.^[^ ^59^ ^]^ Figure 5e shows that the detrimental Cu_Zn_ defects are inhibited in the Li‐doped device, reducing the trap‐assisted recombination. Moreover, the CBO at the front interface is reduced due to the increased the *E*_g_ of CZTSSe absorber after Li doping, which is beneficial to facilitate electronic transport. For Li&Na co‐doped device, although the CBO is slightly enlarged compared with that of Li‐doped device, the bulk acceptors is increased due to the formation of advantageous shallow level Na_Zn_ defects and V_Cu_ (Figure 5f), which is conducive to the enhancement of device performance. Therefore, the performances of the flexible devices are significantly elevated by the synergistic effect of Li doping and Na doping.

### 2.5 Performances of Large‐Area Flexible Devices

**Figure 6:**
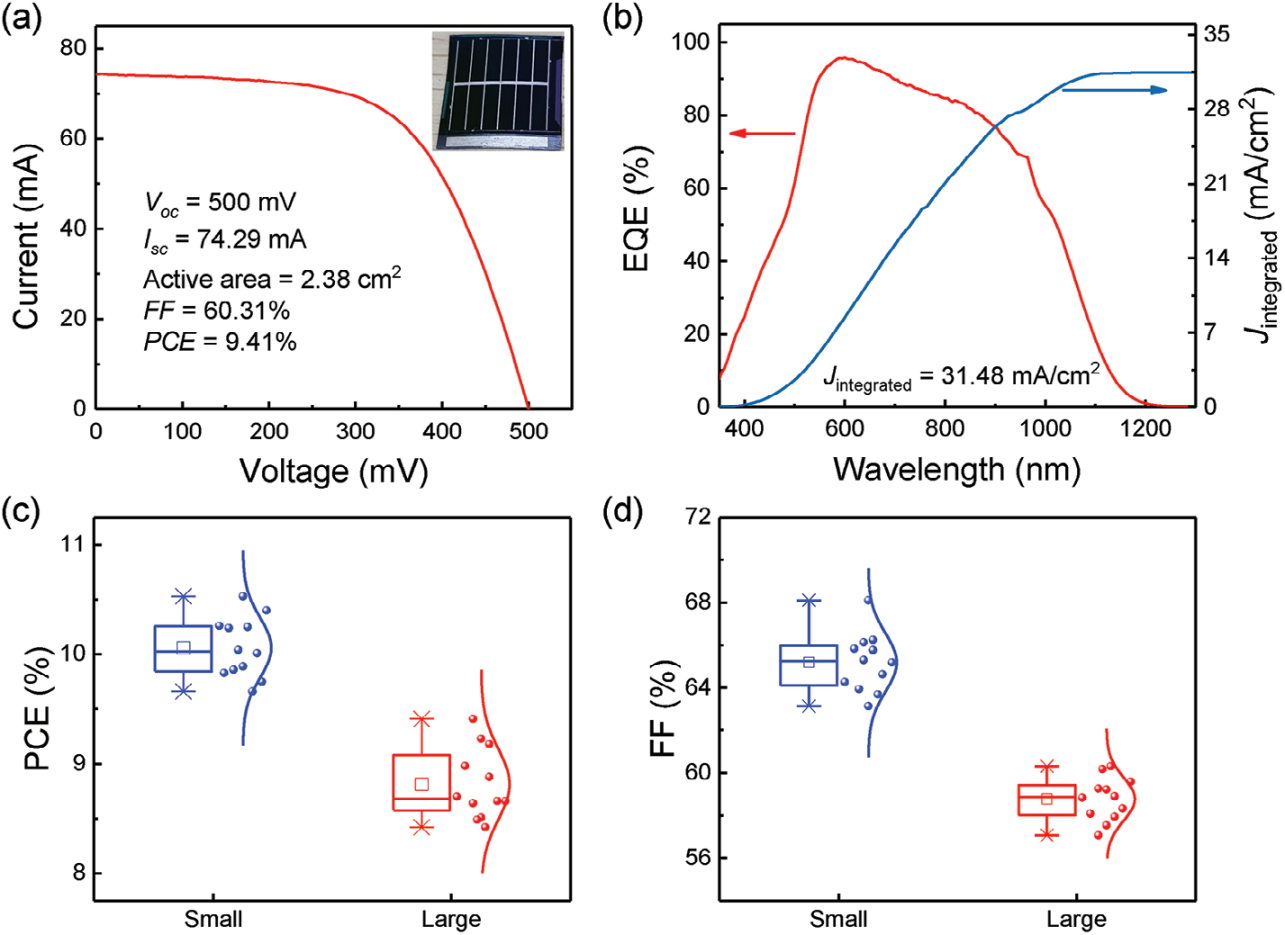
a) The *J*–*V* curve and b) EQE spectra of the Li&Na co‐doped flexible CZTSSe device with an active area of 2.38 cm^2^. The inset in (a) is a photo of large area Li&Na co‐doped solar cell on a 20 × 20 mm flexible Mo foil. The statistical performance box plots for the small‐area (0.205 cm^2^) and large‐area (2.38 cm^2^) flexible devices: c) *PCE* and d) *FF*.

Benefiting from the higher *PCE* obtained by Li&Na co‐doping (Figure 3a), we fabricated the efficient large‐area flexible devices. The *J*–*V* and EQE curves of the large area flexible CZTSSe device with an active area of 2.38 cm^2^ are shown in **Figure** **6**a,b. The champion device achieves a *PCE* of 9.41% with a *V*_oc_ of 500 mV, a short circuit current (*I*_sc_) of 74.29 mA, and an *FF* of 63.6%. That is the highest *PCE* of flexible CZTSSe solar cells with over 2 cm^2^ area reported so far.^[^ ^60^ ^]^ The integrated *J*_sc_ of the large area device extracted from the EQE data is 31.48 mA cm^−2^, according to that (31.21 mA cm^−2^) of the *J*–*V* curves. The statistical performance parameters of the small area (0.205cm^2^) and large area devices are compared as shown in Figure 6c,d and Figure S5 (Supporting Information). When the device area is increased 11.6 times, the average *PCE* and *FF* decline by ≈12.43% and 9.85%, while the average *V*_oc_ and *J*_sc_ slightly reduce by ≈0.43% and 2.42%. This indicates that the device *PCE* of enlarging the device area is mainly restricted by its *FF* parameter. Efficient large‐area flexible devices achieved by the Li&Na co‐doped strategy will promote the development of the CZTSSe solar cells industry.

## 3 Conclusion

In summary, flexible CZTSSe solar cells achieve a significant improvement in the *PCE* via the synergistic effect of Li&Na co‐doping. The Li and Na ions are uniformly incorporated into the CZTSSe films by the solution method with the acetates (LiAc and NaAc) that are cheap, soluble, safe, and non‐toxic are as additives. The results demonstrate that Li ions can be incorporated into the host lattice and widen the *E*_g_ from 1.100 to 1.160 eV, and Na doping can thin the fine grains layer of CZTSSe film and effectively reduce the *V*_oc,def_ by 27 mV. The effect mechanism of Li and Na on the CZTSSe film and its device performances is minutely investigated. It turns out that, in CZTSSe films, Li doping decreases harmful Cu_Zn_ defects concentration from 4.06 × 10^15^ to 3.73 × 10^15^ cm^−3^ and Na doping increases shallow Na_Zn_ defects concentration from 2.98 × 10^14^ to 4.08 × 10^14^ cm^−3^. Furthermore, Li&Na co‐doping reduces the interfacial defects by one order of magnitude (from 10^15^ to 10^14^ cm^−3^). Ultimately, the champion device with Li&Na co‐doping achieves a *PCE* of 10.53% with a certified 10.12%. In addition, we also obtained a large‐area (2.38 cm^2^) flexible CZTSSe device with 9.41% *PCE* due to the excellent synergistic effect of Li&Na co‐doping. The cooperative strategy of Li&Na co‐doping provides a new method for regulating defects and thus enhancing the performances of flexible CZTSSe solar cells.

## 4 Experimental Section

### Materials

Copper (Cu, 99.9%) powder was purchased from Macklin. Zinc (Zn, 99.9%) powder, sulfur (S, 99.9%) powder, 2‐methoxyethanol (C_3_H_8_O_2_, AR), and cadmium sulfate (CdSO_4_, 99%) were purchased from Aladdin. Tin (Sn, 99.8%) powder, selenium (Se, 99%) powder, 1,2‐ethanedithiol (C_2_H_6_S_2_, 98%), ethylenediamine (C_2_H_8_N_2_, 99%), thioglycolic acid (C_2_H_4_O_2_S, 99%), ethanolamine (C_2_H_7_NO, 99.8%) and thiourea (CH_4_N_2_S, 99%) were purchased from Alfa Aesar. Ammonium hydroxide (NH_4_OH, 25%) was purchased from XilongChemical. All chemicals were used directly without further refinement.

### Preparation of Precursor Solution

First, 1.1 mmol Cu, 0.76 mmol Zn, 0.72 mmol Sn, 2.7 mmol S, and 0.3 mmol Se powders were added into a mixed solvent made up of C_2_H_6_S_2_ (0.5 mL) and C_2_H_8_N_2_ (5 mL), which was magnetically stirred for 90 min at 70 ◦C to obtain a clear solution. Then, 1 mL stabilizer made up of C_2_H_7_NO, C_2_H_4_O_2_S, and C_3_H_8_O_2_ was added to the mixed solution and continued spinning until the solution was golden yellow. Those steps were performed in the air. The Li‐doped, Na‐doped, and Li&Na co‐doped CZTSSe precursor solutions were prepared as follows. LiAC and NaAc were simultaneously added to the above solution after it was stirred for 60 min, then continued to be stirred for 30 min and added stabilizer. The Step to add LiAC and NaAc was completed in an Ar‐filled glove box due to their hygroscopy. The Li‐doped CZTSSe precursor solutions were designed: Li/(Cu+Zn+Sn) = 0, 5%, 10%, 15%. The Na‐doped CZTSSe precursor solutions were designed: Na/(Cu+Zn+Sn) = 0, 1%, 2%. The Li&Na co‐doped CZTSSe precursor solutions were designed: Li/(Cu+Zn+Sn) = 10% and Na/(Cu+Zn+Sn) = 1%, 2%.

### Fabrication of CZTSSe Films and Flexible Devices

The CZTSSe absorbers were prepared by spin‐coating method. The CZTSSe precursor solutions were transferred to an Ar‐filled glove box and then spun and coated on the cleaned Mo foils (0.05 mm) followed by a sintering process at 300 °C for 90 s, which was repeated nine times to obtain CZTSSe precursor films(≈2 µm). Finally, the precursor films were selenized at 550 °C for 900 s under an N_2_ atmosphere to obtain crystallographic CZTSSe absorbers. Subsequently, the flexible devices were assembled according to the structure of Mo foil/CZTSSe/CdS/ITO/Ag/MgF_2_. The CdS film of 60 nm thickness was deposited on the absorber by chemical bath approach (CBD). Following this, the ITO film of 250 nm thickness was deposited on the CdS layer by RF magnetron sputtering with low power. Finally, the Ag grid electrode of 500 nm thickness and MgF_2_ film of 80 nm thickness were orderly deposited on the ITO film by the thermal evaporation method. The flexible device with an area of ≈4 cm^2^ was mechanically divided into nine small parts, whose active area was ≈0.205 cm^2^. The active area of a large‐area flexible device was ≈2.38 cm^2^. The device area was determined according to the electrode size obtained under the microscope.

### Characterizations

The micro‐morphologies and crystal structures of absorbers were observed by an SEM (Helios G4 CX) and an XRD (Philips), respectively. The depth compositional profiles of films were analyzed by an ION TOF‐SIMS5 system, where the positive ions in the films were detected by Bi^+^ used as primary ions. The *J‐V* data were collected by a Keithley 2400 source meter under standard illumination (AM 1.5G, 100 mW cm^−2^). The light intensity was calibrated with a standard Si solar cell before the test. The solar cells were tested in the air. The *J‐* *V* curves were obtained in the forward direction with a range from −0.1 to 0.52 V. The EQE spectra were measured by the CROWNTECH CT‐SC‐T QE system. The TDC characterizations were characterized by Keithley 4200 (USA) at the temperature of 100 to 330 K. The AS characterizations were performed by a semiconductor characterization system (Fs‐Pro, Hong Kong). The *C*‐*V* and DLCP characterizations were conducted by Keithley 4200 at a frequency of 50 kHz. The EIS was carried out using an electrochemical workstation (Bio‐Logic SAS, VPS) under a DC bias voltage of 0.3 V and dark (1 Hz to 1 MHz).

## Conflict of Interest

The authors declare no conflict of interest.

## Author Contributions

S.C., H.D., and Q.S. conceived the idea and designed the overall experiments and measurement methods. Q.S. and C.S. contributed to the device fabrications and optimizations. W.X. and Y.L. contributed to characterizations and discussions. S.C., C.Z., J.W., and Q.Z. contributed to discussion and data analysis. Q.S., H.D., and S.C. mainly wrote and revised the manuscript. All authors discussed the results and commented on the manuscript.

## Supporting information

Supporting Information

## Data Availability

The data that support the findings of this study are available from the corresponding author upon reasonable request.
