# Supplementary material for: Defect Synergistic Regulations of Li&Na Co‐Doped Flexible Cu2ZnSn(S,Se)4 Solar Cells Achieving over 10% Certified Efficiency
Source: Adv Sci (Weinh). 2023 Dec 6;11(6):2306740. doi: 10.1002/advs.202306740 (PMC10853737; doi:10.1002/advs.202306740)
Supplement: Supplementary file 1 — Supporting Information [file ADVS-11-2306740-s001.pdf]

ADVANCED SCIENCE
Open Access

Supporting Information

for *Adv. Sci.*, DOI 10.1002/advs.202306740

Defect Synergistic Regulations of Li&Na Co-Doped Flexible $Cu_2ZnSn(S,Se)_4$ Solar Cells Achieving over 10% Certified Efficiency

Quanzhen Sun, Chen Shi, Weihao Xie, Yifan Li, Caixia Zhang, Jionghua Wu, Qiao Zheng, Hui Deng and Shuying Cheng**

Supporting Information

Defect synergistic regulations of Li&Na co-doped flexible $Cu_2ZnSn(S,Se)_4$ solar cells achieving over 10% certified efficiency

Quanzhen Sun, Chen Shi, Weihao Xie, Yifan Li, Caixia Zhang, Jionghua Wu, Qiao Zheng, Hui Deng, and Shuying Cheng**

Q.Z. Sun, C. Shi, W.H. Xie, Y.F. Li, C.X. Zhang, J.H. Wu, Q. Zheng, H. Deng, S.Y. Cheng
Institute of Micro-Nano Devices and Solar Cells
College of Physics and Information Engineering
Fuzhou University
Fuzhou, 350108, P. R. China.
Email: sycheng@fzu.edu.cn (S. Cheng); denghui@fzu.edu.cn (H. Deng)

Q.Z. Sun, C. Shi, W.H. Xie, Y.F. Li, C.X. Zhang, J.H. Wu, H. Deng, S.Y. Cheng
Fujian Science & Technology Innovation Laboratory for Optoelectronic Information of China
Fuzhou, Fujian 350108, P. R. China

Q. Zheng, S. Y. Cheng
Jiangsu Collaborative Innovation Center of Photovoltaic Science and Engineering
Changzhou 213164, P. R. China.

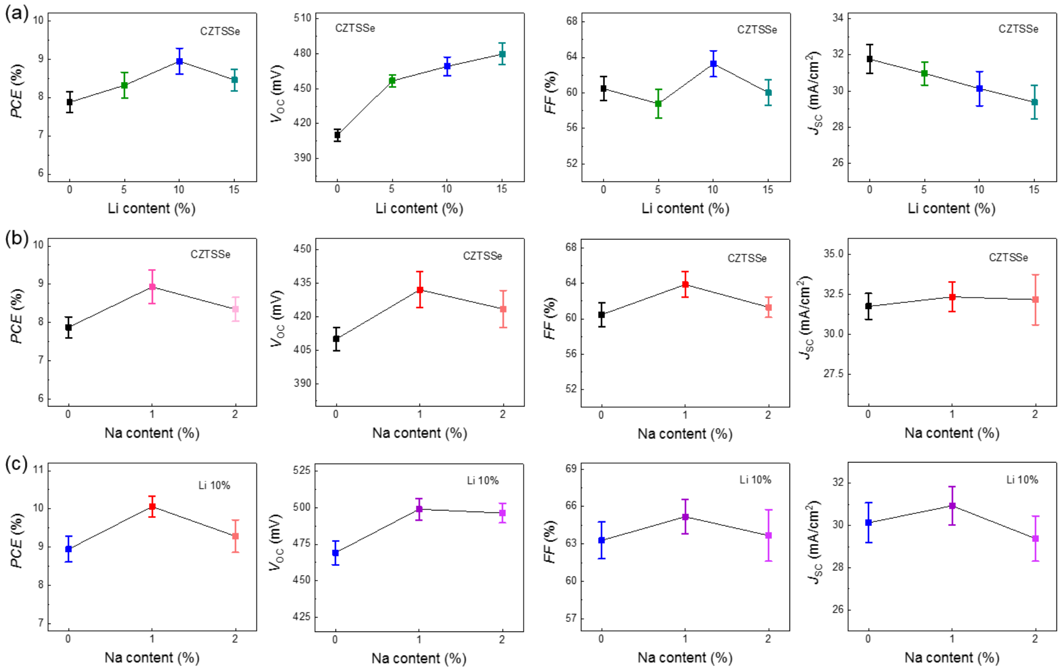

Figure S1. The performance statistics of flexible CZTSSe solar cells with (a) different Li/(Cu+Zn+Sn) ratio, (b) different Na/(Cu+Zn+Sn) ratio and (c) different Na/(Cu+Zn+Sn) ratio based on the optimal Li/(Cu+Zn+Sn).

Table S1. The elemental composition (atomic ratio) of the different absorbers measured by EDS.

**Sample**	**Na (%)**	**Cu (%)**	**Zn (%)**	**Sn (%)**	**Na/(Cu+Zn+Sn)**	**S (%)**	**Se (%)**	**S/(S+Se)**
**undoped**	0.06	14.12	10.39	10.32	0.0017	4.15	60.95	0.0637
**Li-doped**	0	15.13	11.18	10.86	0	4.87	57.06	0.0775
**Na-doped**	0.37	17.74	12.08	11.36	0.0090	3.97	54.48	0.0679
**Li&Na co-doped**	0.48	17.09	18.18	11.31	0.0103	4.68	48.25	0.0884
**Li&Na co-doped(Pre)**	0.33	12.03	9.42	9.96	0.0105	63.37	4.88	0.9285

Table S2. Summary of the average photovoltaic parameters with standard deviations for flexible CZTSSe devices.

**Solar cell**	V_{oc} **(mV)**	J_{sc} **(mA/cm^2)**	***FF*** **(%)**	***PCE*** **(%)**
**undoped**	410±5.2	31.76±0.80	60.47±1.35	7.78±0.27
**Li-doped**	469±8.1	30.13±0.94	63.29±1.48	8.94±0.34
**Na-doped**	432±8.0	32.35±0.94	63.87±1.43	8.93±0.44
**Li&Na co-doped**	499±7.2	30.94±0.90	65.19±1.36	10.06±0.27

福建省计量科学研究院
FUJIAN METROLOGY INSTITUTE
（国家光伏产业计量测试中心）
National PV Industry Measurement and Testing Center

中国认可 国际互认 检测 TESTING CNAS L0131

检 测 报 告
Test Report

报告编号：22Q3-00171
Report No.

客户名称 Name of Customer	College of Physics and Information Engineering, Institute of Micro-Nano Devices and Solar Cells, Fuzhou University
联络信息 Contact Information	No. 2, Xueyuan Road, New District, Fuzhou University, Fuzhou, Fujian Province, China (PR)
物品名称 Name of Items	Flexible CZTSSe solar cell
型号/规格 Type /Specification	Mo foil-based CZTSSe/CdS solar cell
物品编号 Items No	FZU202207-Y3
制造厂商 Manufacturer	College of Physics and Information Engineering, Institute of Micro-Nano Devices and Solar Cells, Fuzhou University
物品接收日期 Items Receipt Date	2022-08-09
检测日期 Test Date	2022-08-09

（盖章处）
Stamp

批准人 Approved by 黎健生
核验员 Checked by 何翔
检测员 Test by 陈彩云

发布日期 Date of Report 2022 年 Year 08 月 month 11 日 Day

扫一扫 查真伪

本院/本中心地址：福州市屏东路9-3号 Address：9-3 Pingdong Road,Fuzhou,China
电话：0591-87845050 Telephone
传真：0591-87808417 Fax
邮编：350003 Post Code
网址：www.fjjl.net Web Site
咨询电话：0591-87845050 Inquire line
投诉电话：0591-87823025 Complaint Tel

未经本院/本中心书面批准，部分复制采用本报告内容无效。
Partly using this Report will not be admitted unless allowed by FMI/ Center.

1. 检测机构说明：
Testing institutions that

本院为国家法定计量检定机构，国家光伏产业计量测试中心依托本院检测技术开展检测。本院/本中心质量管理体系符合 GB/T 27025（ISO/IEC 17025，IDT）标准要求。

The institute is a national legal metrological institution. National PV Industry Measurement and Testing Center carrys out testing relying on the institute's testing technology. The Center's quality management system meets the requirements of GB/T 27025（ISO/IEC 17025，IDT）standard.

2. 本次检测所依据的检测方法（代号及名称）：
Reference documents from the test (code,name)

IEC 60904-1-2020 光伏器件-第一部分：光伏电流-电压特性的测量;IEC 60904-8:2014 光伏器件-第 8 部分光伏器件的光谱响应度测量

3. 本次检测所使用的主要测量仪器：
Measurement standards used in this test

仪器名称 Name	仪器编号 Number	测量范围 Measuring Range	不确定度/或准确度等级/或最大允许误差 Uncertainty or Accuracy Class or Maximum Permissible Error	溯源机构名称/证书编号 Name of traceability institution/Certificate No.	有效期限 Due date
源表	10807C00878-2	电流：-10 μA~1A；电压：20mV~20V	测量：DCV:U_{rel}=0.05%，k=2； DCI：U_{rel}=0.05%，k=2 输出：DCV:U_{rel}=0.05%，k=2； DCI：U_{rel}=0.05%，k=2	福建计量院 22D2-01826	2023-04-13
太阳模拟器	2015-006	(300～1200) nm；(800～1200) W/m^2	光谱匹配度(300～310) nm:U_{rel}=7.4% (k=2);(310～400) nm:U_{rel}=6.4% (k=2);(400～1200) nm:U_{rel}=5.5% (k=2);辐照度比 U_{rel}=1.2% (k=2)	福建计量院 22Q2-00720	2023-06-16
WPVS 单晶硅标准电池	015-2014	(300～1200) nm	U_{rel}=1.3% (k=2)	中国计量院 GXgf2021-10725	2023-04-05
Si 光电探测器	Si-2	(300～1100)nm	(300～400)nm U_{rel}=1.8%～1.7% (k=2)；(400～450)nm U_{rel}=1.7%～1.3% (k=2)；(450～1000)nm U_{rel}=1.3%～1.2% (k=2)；(1000～1100)nm U_{rel}=1.2%～1.7% (k=2)	中国计量院 GXgf2021-10903	2023-03-24
Ge 光电探测器	Ge-2	(900~1600)nm	(900～1050) nm U_{rel}=1.2%～1.7% (k=2);(1050～1600) nm U_{rel}=1.7%～2.0% (k=2)	中国计量院 GXgf2021-10657	2023-03-28

4. 检测地点及环境条件：
Location and environmental condition for the test

地点：Room 108, Building 4, MinHou Scientific Research Base
Location

温度：25.4 ℃ Temperature　　相对湿度：45 % Relative Humidity　　其它：/ Others

5. 备注：/
Note

检测结果/说明：
Results of Test and additional explanation.

1 Standard Test Condition (STC)：Total Irradiance：1000 W/m^2
Temperature：25.0 ℃
Spectral Distribution：AM1.5G

2 Measurement Data and I-V/P-V Curves under STC

Forward Scan

I_{sc} (mA)	V_{oc} (V)	I_{MPP} (mA)	V_{MPP} (V)	P_{MPP} (mW)	FF (%)	η (%)
6.375	0.4933	5.368	0.3788	2.033	64.65	10.04

Reverse Scan

I_{sc} (mA)	V_{oc} (V)	I_{MPP} (mA)	V_{MPP} (V)	P_{MPP} (mW)	FF (%)	η (%)
6.377	0.4951	5.370	0.3810	2.050	64.93	10.12

Mismatch factor: 1.017

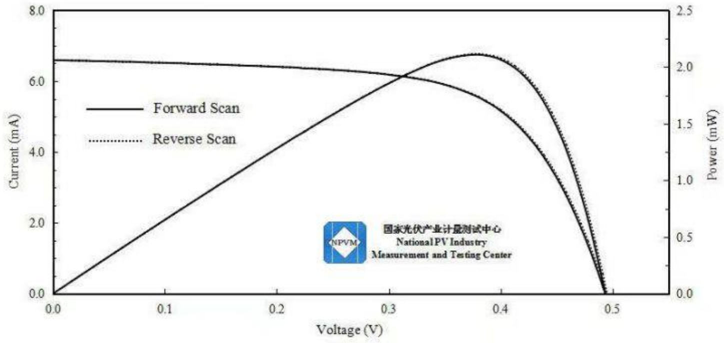

Figure 1. I-V and P-V characteristic curves of the measured sample under STC

检测结果/说明：
Results of Test and additional explanation.

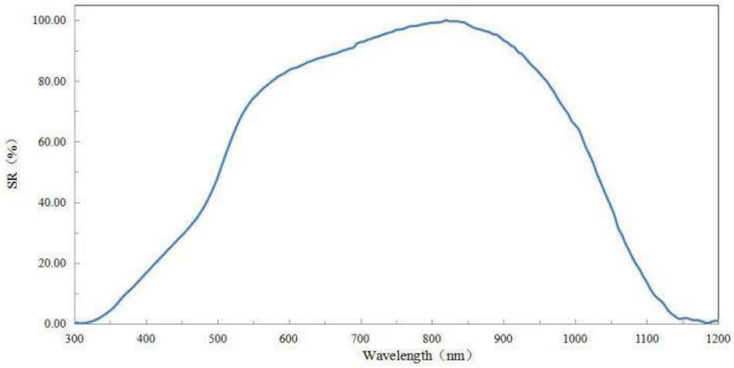

Figure 2. Relative spectral responsivity curve of the measured sample

4 Pictures of the Measured Sample

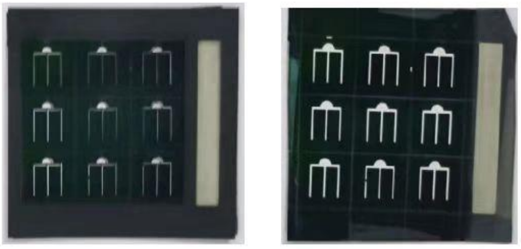

Figure 3.Mask used during test and obverse side of the sample

福建省计量科学研究院
FUJIAN METROLOGY INSTITUTE
（国家光伏产业计量测试中心）
National PV Industry Measurement and Testing Center

报告编号：22Q3-00171
Report No.

检测结果/说明：
Results of Test and additional explanation.

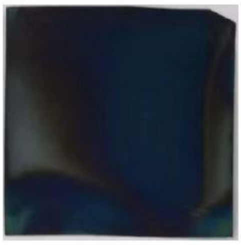

Figure 4. Reverse side of the measured sample

Uncertainty of measurement results:
Short-Circuit Current: U_{rel}=1.4% (k=2); Open-Circuit Voltage: U_{rel}=1.0% (k=2);
Maximum Power: U_{rel}=2.2% (k=2); Efficiency: U_{rel}=2.2% (k=2); Fill Factor: U_{rel}=3.2% (k=2).

Relative Spectral Responsivity:
（300~400）nm: U_{rel} = 2.2% (k=2);
（400~1100）nm: U_{rel} = 1.8% (k=2);
（1100~1200）nm: U_{rel} = 2.6% (k=2).

说明： The designated illuminated area of the measured sample was 0.2025 cm^2 .
Explanation

Testing Method (Code and Name) for This Test
IEC 60904-1: 2020 Photovoltaic devices- Part 1: Measurement of photovoltaic current-voltage characteristics
IEC 60904-8: 2014 Photovoltaic devices- Part 8: Measurement of spectral responsivity of a photovoltaic (PV) device

检测报告续页专用
Continued page of test report

第 7 页/共 8 页
Page of Pages

Figure S2. The certified results of the Li&Na co-doped flexible CZTSSe solar cell measured at by Fujian Metrology Institute/National PV Industry Measurement and Testing Center (NPVM). The certified illuminated area is 0.2025 cm^2 , and certified *PCE* is 10.12% (V_{OC} = 0.4951 V, J_{SC} = 31.49 mA/cm^2, FF = 64.93%).

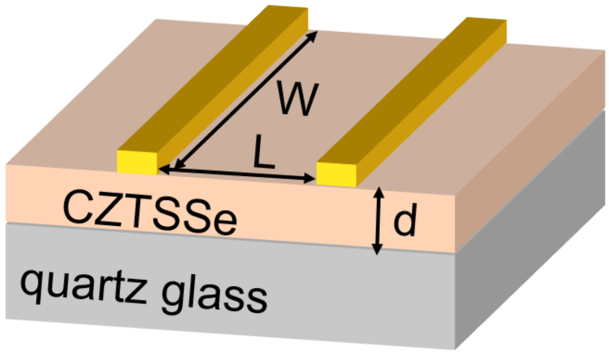

Figure S3. Schematic diagram of the sample structure used for the TDC measurement. There are no alkali metal ions in the quartz glass. The values of W, L and d are 15000, 200 and 2 μm, respectively. The quartz glasses without alkali metal ions ensure that the Li and Na ions in absorbers originate only from the precursor solution.

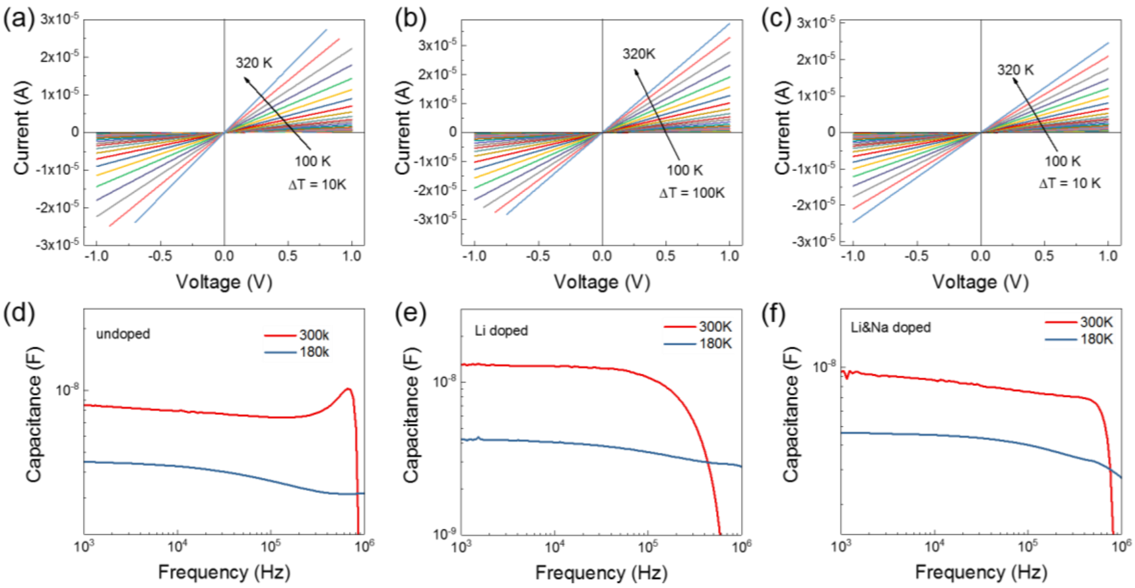

Figure S4. The *I-V* curves of (a) undoped, (b) Li-doped and (c) Li &Na co-doped CZTSSe films in the temperature range of 100-320 K. The *C-F* curves of (d) undoped, (e) Li-doped and Li&Na co-doped flexible CZTSSe solar cells at 180K and 300K.

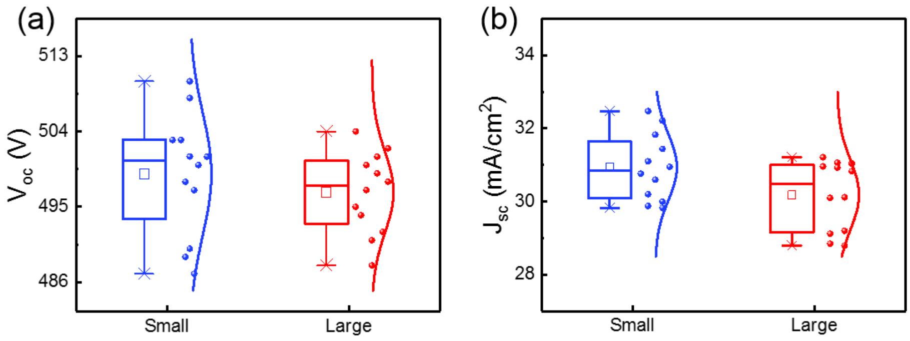

Figure S5. The statistical performance box plots for the small (0.205 cm^2) and large (2.38 cm^2) area Li&Na co-doped flexible CZTSSe devices: (a) V_{oc} and (b) J_{sc}.
